# Verrucacrylic Acids A and B From a Marine Sponge‐derived *Verrucosispora* sp. FIM06025

**DOI:** 10.1002/cbdv.71788

**Published:** 2026-09-30

**Authors:** Minghong Chen, Chengzeng Zhou, Ru Lin, Yuan Yuan, Jian Zhou

**Affiliations:** ^1^ Fujian Provincial Key Laboratory of Screening for Novel Microbial Products Fujian Institute of Microbiology Fuzhou People's Republic of China

**Keywords:** cytotoxic activity, secondary metabolites, structural identification, *Verrucosispora* sp

## Abstract

*Verrucosispora* sp. FIM06025 was an actinomycete isolated from marine sponge sample collected from the East China Sea. Three new compounds, including two rare cyclopropane‐containing compounds, verrucacrylic acids A and B (**1** and **2**), and a new amino acid derivative, methyl *N*‐[(2*S*)‐2‐methylbutanoyl]‐*D*‐tyrosinate (**3**), were isolated from *Verrucosispora* sp. FIM06025. Compounds **1–3** were structurally elucidated on the basis of extensive spectroscopic data analysis using 1D‐ and 2D‐NMR, ECD, and HRESIMS. At a concentration of 100 µM, compounds **1** and **2** exhibited inhibitory rates of 28.6% and 45.2% against T47D cells, respectively, and 30.3% and 47.1% against HL60 cells, respectively.

## Graphical abstract

Three new compounds, including two rare cyclopropane‐containing compounds, verrucacrylic acids A and B (**1** and **2**), and a new amino acid derivative (**3**), were isolated from *Verrucosispora* sp. FIM06025, **1** and **2** exhibited weak cytotoxic activity against HL‐60 and T47D.

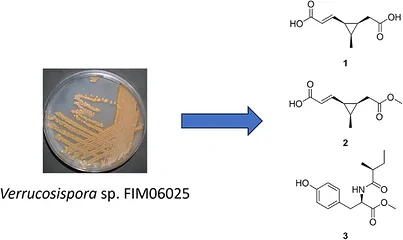

## 1 Introduction

Marine‐derived microorganisms represent a prolific source of structurally unique secondary metabolites with significant pharmacological potential [1]. Among actinomycetes, *Streptomyces* has been the most extensively investigated genus; however, its capacity to yield novel chemical scaffolds has markedly declined over the past decade [2]. In contrast, less‐studied rare actinomycetes—such as *Verrucosispora* and *Micromonospora—*offer enhanced prospects for discovering antibiotics with unprecedented structures, owing to their poorly characterized genetic and physiological traits [3, 4].

The genus *Verrucosispora* [5] has recently garnered considerable interest, as it has proven to be a prolific producer of diverse bioactive molecules, including abyssomicins [6, 7], proximicins [8], gifhornenolones [9], thiocoraline A [10], butrepyrazinone [11], salicylic acid derivative [12], alkaloids [13, 14], and kendomycin [15]. This chemical diversity is consistent with genomic analyses of *Verrucosispora* sp., which have revealed multiple biosynthetic gene clusters, including polyketide synthases (PKSs), nonribosomal peptide synthetases (NRPSs), and terpene synthases [3, 12]. Cyclopropane‐containing natural products are relatively rare in nature, with most reported examples originating from terpenoids, fatty acids, and some alkaloids. Their unique strained ring system often contributes to significant biological activities, making them attractive targets for drug discovery. The isolation of cyclopropane‐bearing compounds from marine actinomycetes, however, remains uncommon. In this paper, the fermentation of the strain *Verrucosispora* sp. FIM06025 and the isolation, purification, physicochemical characterization, and cytotoxic activity evaluation of compounds **1–3** were presented.

## 2 Results and Discussion

Verrucacrylic acid A (**1**) isolated as a primrose yellow oil, was assigned a molecular formula C_9_H_12_O_4_ on the basis of HRESIMS analysis *m*/*z* 207.0633 [M + Na]^+^ (Figure S7). The ^1^H NMR of **1** exhibited two olefinic protons at *δ*_H_ 6.77 (dd, *J* = 15.2, 10.9 Hz, H‐3), 5.95 (d, *J* = 15.2 Hz, H‐2); three methine groups at *δ*_H_ 1.78 (m, H‐4), 1.59 (p, *J* = 8.3 Hz, H‐5), 1.45 (ddt, *J* = 15.4, 8.8, 6.9 Hz, H‐8); one methylene group at *δ*_H_ 2.45 (m, H‐6); and one methyl group at *δ*_H_ 1.11 (d, *J* = 6.7 Hz, H‐9) (Table 1 and Figure S1). The ^13^C NMR and HSQC contained two carbonyl signals at *δ*_C_ 168.4 and 175.2, two alkene resonances at *δ*_C_ 121.9 and 147.4 (Figures S2 and S3). The correlations of H‐2/H‐3/H‐4/H‐5/H‐6, H‐9/H‐8/H‐4, H‐8/H‐5 in the COSY (Figure S4) revealed the presence of an unusual trisubstituted cyclopropane skeleton in **1**. This was supported by the correlations of HMBC (Figure S5) from H_3_‐9 (*δ*_H_ 1.11) to C‐4 (*δ*_C_ 21.7), C‐5 (*δ*_C_ 20.4), C‐8 (*δ*_C_ 18.1); H_2_‐6 (*δ*_H_ 2.45) to C‐4, C‐5, C‐8 (Figure 2). The correlations of HMBC from H_2_‐6 to C‐7 (*δ*_C_ 175.2); H‐2 (*δ*_H_ 5.95), H‐3 (*δ*_H_ 6.77) to C‐1 (*δ*_C_ 168.4) determined that the two carboxyl groups were located at C‐6 and C‐2, respectively. The 4R*, 5S*, and 8R* relative configuration of the three asymmetric carbons was established by analysis of the NOESY correlations of H‐3/H_2_‐6/H_3_‐9 (Figures 3 and S6). Consequently, the absolute configuration of the new compound **1** (Figure 1) was confirmed as 4*R*,5*S*,8*R* by the ECD experiment (Figure 4).

**TABLE 1:** ^1^H NMR (600 MHz) and ^13^C NMR (150 MHz) data for **1–3** in CD_3_OD.

No.	1		2		3	
	*δ*_C_ (ppm), type	*δ*_H_ (mult., *J* in Hz)	*δ*_C_ (ppm), type	*δ*_H_ (mult., *J* in Hz)	*δ*_C_ (ppm), type	*δ*_H_ (mult., *J* in Hz)
1	168.4, C		168.4, C		172.5, C	—
2	121.9, CH	5.95 (d, 15.2)	122.0, CH	5.95 (d, 15.2)	53.9, CH	4.57 (dd, 9.2, 5.6)
3	147.4, CH	6.77 (dd, 15.2, 10.9)	147.2, CH	6.75 (dd, 15.2, 10.9)	36.3, CH_2_	3.03 (dd, 13.9, 5.6) 2.82 (dd, 13.9, 9.3)
4	21.7, CH	1.78 (m)	21.8, CH	1.78 (m)	127.6, C	—
5	20.4, CH	1.59 (p, 8.3)	20.3, CH	1.59 (p, 8.3)	129.9, CH	6.99 (d, 8.5)
6	28.5, CH_2_	2.45 (m)	28.5, CH_2_	2.52 (dd, 16.8, 8.0) 2.47 (dd, 16.8, 7.4)	114.8, CH	6.66 (d, 8.5)
7	175.2, C		173.7, C		156.1, C	
8	18.1, CH	1.45 (ddt, 15.4, 8.8, 6.9)	18.1, CH	1.46 (ddt, 15.4, 8.5, 6.7)	114.8, CH	6.66 (d, 8.5)
9	7.4, CH_3_	1.11 (d, 6.7)	7.4, CH_3_	1.10 (d, 6.7)	129.9, CH	6.99 (d, 8.5)
10			50.9, CH_3_	3.64 (s)	178.1, C	—
11					42.1, CH	2.20 (m)
12					26.9, CH_2_	1.53 (m) 1.32 (m)
13					10.8, CH_3_	0.83 (t, 7.4)
14					16.6, CH_3_	0.94 (d, 6.9)
15					51.2, CH_3_	3.65 (s)

**FIGURE 1:**
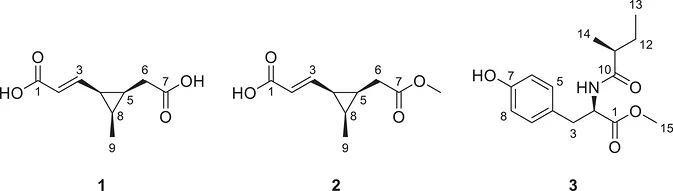
Compounds **1–3** from *Verrucosispora* sp. FIM06025.

The molecular formula of compound **2** was deduced as C_10_H_14_O_4_ from the ion peak at *m*/*z* 221.0786 [M + Na]^+^ (Figure S14) in the HRESIMS. The ^1^H NMR (Table 1 and Figure S8) displayed two olefinic protons at *δ*_H_ 6.75 (dd, *J* = 15.2, 10.9 Hz, H‐3), 5.95 (d, *J* = 15.2 Hz, H‐2); three methine groups at *δ*_H_ 1.78 (m, H‐4), 1.59 (p, *J* = 8.3 Hz, H‐5), 1.46 (ddt, *J* = 15.4, 8.5, 6.7 Hz, H‐8); one methylene group at *δ*_H_ 2.47 (dd, *J* = 16.8, 7.4 Hz, H‐6a) and 2.52 (dd, *J* = 16.8, 8.0 Hz, H‐6b); one methoxy at *δ*_H_ 3.64 (s, H‐10); and one methyl group at *δ*_H_ 1.10 (d, *J* = 6.7 Hz, H‐9). The presence of 10 resonances, including two carbonyl (*δ*_C_ 168.4 and 173.7) and two alkene (*δ*_C_ 122.0 and 147.2) signals, was evident in the ^13^C NMR (Table 1 and Figure S9). Comparison of the ^1^H, ^13^C, and 2D NMR data of **2** (Table 1 and Figures S10–S12) with those of **1** revealed that the presence of another methoxy in **2** was the difference between the two compounds. The correlations of HMBC (Figure S12) from H_2_‐6 (*δ*_H_ 2.47 and 2.52), H_3_‐10 (*δ*_H_ 3.64) to C‐7 (*δ*_C_ 173.7) determined that the methoxy was located at C‐7 (Figure 2). The correlations of H‐3/H_2_‐6/H_3_‐9 were observed in NOESY spectrum (Figure 3 and S13), and it was proposed that the relative configurations of **2** and **1** were the same. The absolute configuration of **2** was unambiguously determined due to the experimental ECD curve matched the curve calculated for (4*R*,5*S*,8*R*)‐**2** (Figure 4).

**FIGURE 2:**
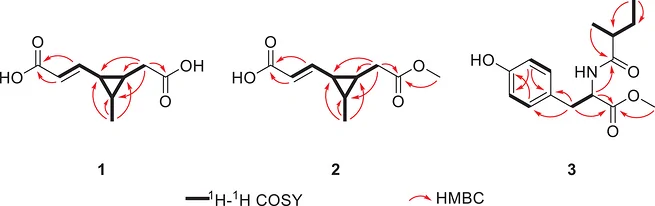
Key ^1^H‐^1^H COSY and HMBC correlations of **1–3**.

**FIGURE 3:**
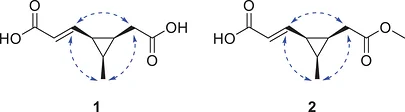
Key NOESY correlations of **1–2**.

**FIGURE 4:**
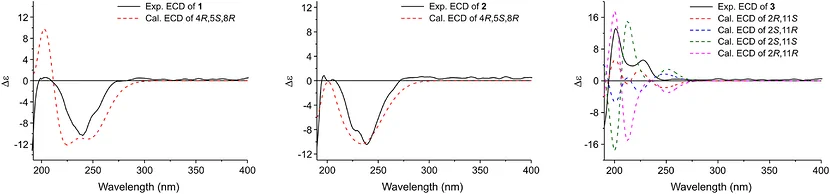
Experimental and calculated ECD spectra of 1–3.

Compound **3** was isolated as a primrose yellow oil with molecular formula C_15_H_21_NO_4_ inferred from its HRESIMS ion at *m*/*z* 280.1544 [M + H]^+^ (Figure S20). The ^1^H NMR spectrum (Table 1 and Figure S15) showed four aromatic protons at *δ*_H_ 6.66 (d, *J* = 8.5 Hz, H‐6, 8), 6.99 (d, *J* = 8.5 Hz, H‐5, 9), two methylene signals at *δ*_H_ 3.03 (dd, *J* = 13.9, 5.6 Hz, H‐3a), 2.82 (dd, *J* = 13.9, 9.3 Hz, H‐3b), 1.53 (m, H‐12a), 1.32 (m, H‐12b), two methine signals at *δ*_H_ 4.57 (dd, *J* = 9.2, 5.6 Hz, H‐2), 2.20 (m, H‐11), one methoxy at *δ*_H_ 3.65 (s, H‐15), and two methyl signals at *δ*_H_ 0.94 (d, *J* = 6.9 Hz, H‐14), 0.83 (t, *J* = 7.4 Hz, H‐13), suggesting that **3** contains a tyrosine residue. This was supported by the ^13^C NMR (Figure S16) and HSQC (Figure S17). The ^1^H‐^1^H COSY correlations (Figure S18) of H_3_‐14/H‐11/H_2_‐12/H_3_‐13 and the HMBC correlations (Figure S19) from H_3_‐14 (*δ*_H_ 0.94), H‐2 (*δ*_H_ 4.57) to C‐10 (*δ*_C_ 178.1), H_3_‐15 (*δ*_H_ 3.65) to C‐1 (*δ*_C_ 172.5) revealed that the 2‐methylbutanoyl group is an *N*‐acyl substituent and the methoxy group is part of a methyl ester at C‐1. By comparing the experimental ECD curve with the calculated curve, the absolute configuration of compound **3** was determined to be 2*R*,11*S* (Figure 4). Thus, the structure of **3** was established as methyl *N*‐[(2*S*)‐2‐methylbutanoyl]‐*D*‐tyrosinate (Figure 1).

All compounds were tested against a panel of human cancer cell lines (HL‐60, ECA‐109, and T47D cell lines). Compounds **1** and **2** demonstrated weak cytotoxicity against the tested cancer cell lines. At a concentration of 100 µM, compounds **1** and **2** exhibited inhibitory rates of 28.6% and 45.2% against T47D cells, respectively, and 30.3% and 47.1% against HL60 cells, respectively. Compound **3** showed negligible activity against all tested cell lines, with inhibition rates of less than 10% at 100 µM. Cisplatin was used as a positive control, showing inhibition rates of 88.6% and 91.3% against T47D and HL60 cells at 25 µM, respectively. The vehicle control (0.1% DMSO) showed no significant inhibition.

## 3 Conclusion

In summary, chemical investigation of the secondary metabolites produced by *Verrucosispora* sp. FIM06025 led to the discovery of three new compounds (**1–3**), further enriching the structural diversity known from this species. The absolute configurations of **1–3** were determined through ECD analysis. All compounds were evaluated for cytotoxicity against HL‐60, ECA‐109, and T47D cell lines; among them, compounds **1** and **2** exhibited weak cytotoxic activity against HL‐60 and T47D.

## 4 Experimental Section

### 4.1 General Experiment Procedures

UV absorption spectra were acquired using a Shimadzu UV‐1800 spectrophotometer, while ECD data were obtained on a JASCO J‐1700 circular dichroism spectrometer. All 1D and 2D NMR measurements were performed on a JEOL 600 MHz instrument. HRESIMS analyses were conducted on an LC/MS Q‐Orbitrap system and an Agilent 6545 Q‐TOF mass spectrometer. Semipreparative HPLC separations were carried out on a Shimadzu LC‐8A system equipped with an Agilent SB‐C18 column (20 × 250 mm, 10 µm).

### 4.2 Actinomycete Strain

The strain FIM06025 was obtained from a marine sponge (*Demospongiae* sp.) collected at ∼5 m depth off the coast of Fujian Province (East China Sea). Its 16S rRNA gene sequence has been deposited in GenBank under accession number EU696750.1. Phylogenetic analysis revealed that strain FIM06025 shares the highest similarity with the type strain *Verrucosispora* sp., thereby confirming its identification as *Verrucosispora* sp. FIM06025 (Figure S21).

### 4.3 Fermentation and Extraction

The *Verrucosispora* sp. FIM06025 was grown on a ISP2 plate at 28°C for 7 days. It was used to inoculate 60 L medium (soluble starch 2.0–4.0wt%, glucose 0.5–1.5wt%, peptones 0.25–0.7wt%, yeast extract 0.25–1.0wt%, CaCO_3_ 0.1–0.3wt%, pH 6.0–8.5). The cultures were fermented at 30°C for 28 days. Crude extract (20 g) was obtained from the fermented material by successive extraction with ethyl acetate (60 L, three times), followed by solvent evaporation.

### 4.4 Isolation

The extract was subjected to silica‐gel column, eluted by DCM‐MeOH, affording subfractions Fr.1‒Fr.15. All 15 subfractions were analyzed by HPLC and TLC. Fractions 6 and 8 were selected for further purification, while the other fractions contained complex mixtures that could not be purified to obtain reportable compounds. The subfraction Fr. 6 was separated using pre‐HPLC eluted by gradient MeOH‐H_2_O (40:60), at 10 mL/min flow rate. As a result, compounds **1** (Rt. 17 min, 5.6 mg) and **2** (Rt. 29 min, 9.1 mg) were obtained. To confirm the natural origin of compound **2**, a targeted LC‐MS analysis of the original crude extract was performed, which confirmed the presence of the [M + Na]^+^ ion of **2**. Compound **3** (Rt. 31 min, 3.2 mg) was purified by pre‐HPLC (MeOH‐H_2_O 20:80‒100:0, 60 min) in Fr. 8. The IR spectra of compounds **1–3** were shown in Figures S22–S24. The purities of compounds **1–3** were determined to be >95% by HPLC analysis (Figures S25–S27).

Verrucacrylic acid A (**1**): primrose yellow oil; ${\left[\alpha \right]}_{D}^{20}$‒0.0018 (*c* 0.05, MeOH); UV (MeOH) *λ*_max_ (log *ε*) 194 (1.26), 237 (2.07) nm; ECD (0.5 mg/mL, MeOH) *λ*_max_ (Δ*ε*), 204 (+0.60), 240 (‒10.36) nm; IR *ν*_max_ 3370, 1699, 1648, 1350, 1266, 1205, 989 cm^−1^; ^1^H and ^13^C NMR (600 MHz, CD_3_OD) data shown in Table 1; HRESIMS *m/z* 207.0633 [M + Na]^+^ (calcd for C_9_H_12_O_4_Na; 207.0628).

Verrucacrylic acid B (**2**): primrose yellow oil; ${\left[\alpha \right]}_{D}^{20}$‒0.0003 (*c* 0.05, MeOH); UV (MeOH) *λ*_max_ (log *ε*) 194 (1.32), 236 (1.90) nm; ECD (0.5 mg/mL, MeOH) *λ*_max_ (Δ*ε*) 197 (+0.84), 205 (+0.19), 228 (−8.21), 239 (−10.47) nm; IR *ν*_max_ 3370, 1699, 1648, 1350, 1266, 1205, 1179, 1165, 989 cm^−1^; ^1^H and ^13^C NMR (600 MHz, CD_3_OD) data shown in Table 1; HRESIMS *m*/*z* 221.0786 [M + Na]^+^ (calcd for C_10_H_14_O_4_Na; 221.0784).

Methyl *N*‐[(2*S*)‐2‐methylbutanoyl]‐*D*‐tyrosinate (**3**): primrose yellow oil; ${\left[\alpha \right]}_{D}^{20}$‒0.0012 (*c* 0.05, MeOH); UV (MeOH) *λ*_max_ (log *ε*) 195 (1.50), 222 (0.35), 269 (0.09) nm; ECD (0.5 mg/mL, MeOH) *λ*_max_ (Δ*ε*) 201 (+13.16), 227 (+5.19) nm; IR *ν*_max_ 2831, 2716, 1632, 1364, 1104, 989 cm^−1^; ^1^H and ^13^C NMR (600 MHz, CD_3_OD) data shown in Table 1; HRESIMS *m/z* 280.1544 [M + H]^+^ (calcd for C_15_H_22_NO_4_; 280.1543).

### 4.5 Cell Culture and Cytotoxicity Assay

Cytotoxic activity was evaluated following a previously established protocol [16]. Briefly, HL‐60 (RRID: CVCL_0002), ECA‐109 (RRID: CVCL_6898), and T47D (RRID: CVCL_0553) cell lines were cultured in RPMI‐1640 medium containing 10% FBS, 100 U/mL penicillin, and 100 µg/mL streptomycin, under a humidified 5% CO_2_ atmosphere at 37°C for 24 h. Cells were then exposed to 100 µM of compounds **1–3** for 48 h, with cisplatin (25 µM) and 0.1% DMSO serving as positive and vehicle controls, respectively. Adherent lines (ECA‐109 and T47D) were seeded in 96‐well plates and allowed to attach for 24 h prior to treatment, whereas suspension cells (HL‐60) were seeded similarly and incubated for 24 h before compound addition. Cell viability was quantified via MTT assay. All tests were run in triplicate, with results expressed as mean ± SD; statistical comparisons were performed using Student's *t*‐test, with significance set at *p* < 0.05.

### 4.6 Calculation of ECD Data

Conformational searches were performed using the MCMM torsional sampling method with the MMFFs force field in Spartan 14. The resulting conformers were re‐optimized at the PBE1PBE/6‐311G(d) level incorporating PCM solvation models via ORCA 5.0.3 [17]. Frequency calculations confirmed that the optimized geometries corresponded to true energy minima. TD‐DFT‐based ECD spectra were computed for these conformers using the ωB97XD/def2‐TZVP basis set, and Boltzmann‐averaged spectra were subsequently generated with SpecDis 1.71 [18].

## Author Contributions

**Minghong Chen**: investigation, writing – original draft. **Chengzeng Zhou**: data curation, conceptualization. **Ru Lin**: resources. **Yuan Yuan**: validation. **Jian Zhou**: writing – review and editing.

## Conflicts of Interest

The authors declare no conflicts of interest.

## Supporting information




**Supporting File**: cbdv71788‐sup‐0001‐SuppMat.docx.

## Data Availability

The data that support the findings of this study are available in the Supporting Information of this article.
