# Supplementary material for: Verrucacrylic Acids A and B From a Marine Sponge‐derived Verrucosispora sp. FIM06025
Source: Chem Biodivers. 2026 Sep 30;23(10):e71788. doi: 10.1002/cbdv.71788 (PMC13626856; doi:10.1002/cbdv.71788)
Supplement: Supplementary file 1 [file CBDV-23-e71788-s001.docx]

Supporting Information

Verrucacrylic acids A and B from marine sponge derived *Verrucosispora* sp. FIM06025

Minghong Chen, Chengzeng Zhou, Ru Lin, Yuan Yuan and Jian Zhou ^*^

**Affiliation**

*Fujian Provincial Key Laboratory of Screening for Novel Microbial Products, Fujian Institute of Microbiology, Fuzhou, 350007, People’s Republic of China*

*Corresponding author. E-mail: zjian503@163.com;

Fax: +86-591-83441193. Tel: +86-591-83475544.

**Table of contents**

Supplementary Figures	1

Figure S1. ^1^H NMR of compound 1 (in CD_3_OD, 600 MHz).	2

Figure S2. ^13^C NMR of compound 1 (in CD_3_OD, 150 MHz).	2

Figure S3. HSQC of compound 1 (in CD_3_OD).	3

Figure S4. COSY of compound 1 (in CD_3_OD).	3

Figure S5. HMBC of compound 1 (in CD_3_OD).	4

Figure S6. NOESY of compound 1 (in CD_3_OD).	4

Figure S7. HRESIMS of compound 1.	5

Figure S8. ^1^H NMR of compound 2 (in CD_3_OD, 600 MHz).	5

Figure S9. ^13^C NMR of compound 2 (in CD_3_OD, 150 MHz).	6

Figure S10. HSQC of compound 2 (in CD_3_OD).	6

Figure S11. COSY of compound 2 (in CD_3_OD).	7

Figure S12. HMBC of compound 2 (in CD_3_OD).	7

Figure S13. NOESY of compound 2 (in CD_3_OD).	8

Figure S14. HRESIMS of compound 2.	8

Figure S15. ^1^H NMR of compound 3 (in CD_3_OD, 600 MHz).	9

Figure S16. ^13^C NMR of compound 3 (in CD_3_OD, 150 MHz).	9

Figure S17. HSQC of compound 3 (in CD_3_OD).	10

Figure S18. COSY of compound 3 (in CD_3_OD).	10

Figure S19. HMBC of compound 3 (in CD_3_OD).	11

Figure S20. HRESIMS of compound 3.	11

Figure S21. The phylogenetic tree of strain FIM06025.	12

Figure S22. IR spectrum of compound 1.	12

Figure S23. IR spectrum of compound 2.	13

Figure S24. IR spectrum of compound 3	13

Figure S25. HPLC purity chromatograms of compound 1	14

Figure S26. HPLC purity chromatograms of compound 2	14

Figure S27. HPLC purity chromatograms of compound 3	15

## **Figure S1.** ^1^H NMR of compound **1** (in CD_3_OD, 600 MHz).

## **Figure S2.** ^13^C NMR of compound **1** (in CD_3_OD, 150 MHz).

## **Figure S3.** HSQC of compound **1** (in CD_3_OD).

**Figure S4.** COSY of compound **1** (in CD_3_OD).

## **Figure S5.** HMBC of compound **1** (in CD_3_OD).

## **Figure S6.** NOESY of compound **1** (in CD_3_OD).

## **Figure S7.** HRESIMS of compound **1**.

## **Figure S8.** ^1^H NMR of compound **2** (in CD_3_OD, 600 MHz).

## **Figure S9.** ^13^C NMR of compound **2** (in CD_3_OD, 150 MHz).

## **Figure S10.** HSQC of compound **2** (in CD_3_OD).

## **Figure S11.** COSY of compound **2** (in CD_3_OD).

## **Figure S12.** HMBC of compound **2** (in CD_3_OD).

## **Figure S13.** NOESY of compound **2** (in CD_3_OD).

## **Figure S14.** HRESIMS of compound **2**.

## **Figure S15.** ^1^H NMR of compound **3** (in CD_3_OD, 600 MHz).

## **Figure S16.** ^13^C NMR of compound **3** (in CD_3_OD, 150 MHz).

## **Figure S17.** HSQC of compound **3** (in CD_3_OD).

**Figure S18.** COSY of compound **3** (in CD_3_OD).

## **Figure S19.** HMBC of compound **3** (in CD_3_OD).

## **Figure S20.** HRESIMS of compound **3**.

**Figure S21.** The phylogenetic tree of strain FIM06025

## **Figure S22.** IR spectrum of compound **1**.

## **Figure S23.** IR spectrum of compound **2**.

## **Figure S24.** IR spectrum of compound **3**.

## **Figure S25.** HPLC purity chromatograms of compound **1**.

## **Figure S26.** HPLC purity chromatograms of compound **2**.

## **Figure S27.** HPLC purity chromatograms of compound **3**.
